# Latent profiles of cyberchondria among nursing interns: the differential mediating role of health anxiety in the relationship between work stress and profile membership

**DOI:** 10.3389/fpubh.2026.1899742

**Published:** 2026-07-27

**Authors:** Jiahao Yuan, Xiangrong Wang, Yong Fang, Huijuan He, Mengying Li, Na Zhang

**Affiliations:** School of Nursing, Hubei University of Chinese Medicine, Wuhan, Hubei, China

**Keywords:** cyberchondria, health anxiety, latent profile analysis, nursing interns, work stress

## Abstract

**Background:**

In the digital health era, the internet has become a primary channel for the public to access health information. However, fragmented and non-professional online content can easily foster cyberchondria. Nursing interns—being a transitional group with foundational medical knowledge yet facing significant clinical practice stress—are particularly vulnerable to cyberchondria. Nevertheless, existing studies have predominantly focused on general university students, with limited in-depth examination of the heterogeneous manifestations of cyberchondria among nursing interns. Therefore, this study aims to identify latent classes of cyberchondria among nursing interns and to examine the associations of work stress with profile membership and the indirect associational pathways of health anxiety consistent with hypothesized mediation pathways.

**Methods:**

A convenience sampling method was used to recruit 538 nursing interns from 14 tertiary Grade-A hospitals in Hubei Province between September and November 2025. To collect relevant data, participants completed a general information questionnaire, the Chinese version of the Short Cyberchondria Scale, the Short Health Anxiety Inventory, and the Nursing Internship Stress Scale. Subsequently, latent profile analysis was employed to identify latent classes of cyberchondria among the nursing interns.

**Results:**

Cyberchondria among nursing interns could be classified into four latent classes: the low cyberchondria-apathetic type (8.7%), the moderate cyberchondria-prudent type (44.2%), the relatively high cyberchondria-irritable type (38.0%), and the high cyberchondria-obsessive type (9.1%). Work stress was negatively associated with membership in the low cyberchondria-apathetic type (β = −0.13, *P* = 0.019), positively associated with membership in the relatively high cyberchondria-irritable type (β = 0.10, *P* = 0.037), and showed no statistically significant direct association on membership in the high cyberchondria-obsessive type (β = −0.04, *P* = 0.382). Health anxiety exhibited differential indirect associational pathways between work stress and three of the four cyberchondria types, with full mediation observed for the high cyberchondria-obsessive type (standardized effect value = 0.105, *P* < 0.001).

**Conclusions:**

There is significant heterogeneity in cyberchondria among nursing interns. Work stress is associated with elevated cyberchondria by increasing health anxiety. Therefore, reducing work stress and implementing interventions that target health anxiety represent effective strategies for reducing cyberchondria in this population.

## 1 Introduction

With the advent of the digital health era, the internet has become the most convenient channel for the public to access health-related information (1). According to the 57th Statistical Report on China's Internet Development, released by the China Internet Network Information Center (2), as of December 2025, the number of internet users in China had reached 1.125 billion, and the internet penetration rate had reached 80.1%. The widespread practice of seeking online health information has led individuals, when experiencing discomfort, to prioritize searching for disease information related to their symptoms through search engines rather than directly seeking professional medical help. Previous research has identified a “symptom escalation” phenomenon in this behavior: individuals conducting searches tend to associate common, potentially benign symptoms with descriptions of more severe illnesses. As they search for information, their health concerns gradually intensify (3). Moreover, given the characteristics of online health information—such as diverse sources, uneven quality, and lack of professional review—the public is more likely to develop cognitive biases when accessing and evaluating such information, which may in turn be associated with the onset or elevation of cyberchondria.

Cyberchondria refers to a condition where individuals, driven by anxiety and distress regarding their own health, conduct excessive or repeated online searches for health-related information. This behavior paradoxically amplifies their anxiety or distress, leading to heightened stress, excessive worry, and unnecessary medical expenditures (4, 5). Subsequently, scholars have continued to deepen their understanding of cyberchondria: McElroy and Shevlin (6) developed the Cyberchondria Severity Scale, operationalizing it as a measurable and multidimensional construct. Fergus (7) further confirmed the unique association between cyberchondria and health anxiety, noting that cyberchondria is not merely an extension of health anxiety but possesses distinct psychological and behavioral characteristics. Cyberchondria is now recognized as an increasingly prominent global mental health concern. Research indicates that approximately 75% of people worldwide search the internet for health-related information concerning their own health (8). A cross-sectional survey among Chinese university students found that 57.6% exhibited symptoms of cyberchondria (9), suggesting that the prevalence of cyberchondria among young populations warrants serious attention.

When examining the mechanisms underlying the development of cyberchondria, health anxiety is considered a core proximal driver (10). Health anxiety refers to a psychological state characterized by excessive worry and fear about one's own health status, involving heightened attention to bodily sensations, catastrophic misinterpretation of illness-related information, and a recurrent need for reassurance (11). Muse et al. (12) found that health anxiety significantly predicts both the frequency and duration of online health information seeking, particularly among individuals with poor health information discrimination skills. Santoro et al. (13) further confirmed that health anxiety mediates the relationship between somatic symptoms and cyberchondria, suggesting its critical role in the onset and maintenance of cyberchondria.

Nursing interns, as a transitional group moving from academic training to clinical practice, possess foundational professional knowledge that has not yet been systematically consolidated: they have completed formal medical education and acquired basic knowledge of diseases and symptom recognition, yet their clinical experience remains limited, and their capacity for differential diagnosis is underdeveloped. This “semi-professional” status places them in a unique position—unlike the general public, they cannot rely solely on intuition when assessing their own health; nor can they draw upon extensive clinical expertise like experienced nurses to make accurate judgments. Consequently, they are more likely to turn to online searches to verify and supplement their health-related self-assessments (14). Meanwhile, nursing interns face multiple stressors in clinical practice, including high workloads, elevated risk of procedural errors, and diminished professional identity (15, 16). These stressors not only impair their mental wellbeing but may also be linked to elevated cyberchondria by heightening health anxiety. However, although existing studies have examined cyberchondria among medical students, nursing interns—as a distinct transitional clinical cohort—have received comparatively little attention (17, 18). In particular, latent profile analysis has not yet been applied to identify empirically derived subtypes of cyberchondria in this population, hindering the development of precise, subgroup-specific interventions.

As a distal environmental factor, work stress may also indirectly influence cyberchondria through health anxiety. According to the transactional stress model proposed by Lazarus and Folkman (19), when individuals perceive that environmental demands exceed their coping resources, they undergo stress appraisal and experience negative emotional reactions. Health anxiety is an emotional manifestation of an individual's overestimation of health threats in stressful situations. Stressors in the clinical work environment—such as high workload, risk of operational errors, and threat of occupational exposure (15)—may continuously activate individuals' perception of health threats, increase their sensitivity to bodily sensations, and thereby elevate health anxiety levels. Błachnio et al. (20) demonstrated that stress appraisal can exacerbate cyberchondria through rumination as a mediating mechanism. However, existing research has predominantly focused on general adult or university student populations, with insufficient attention to nursing students during their clinical internships. To effectively identify subgroups with distinct characteristics, this study employed latent profile analysis—a person-centered statistical method that classifies participants into latent classes based on patterns of continuous variables, thereby revealing population heterogeneity (21).

In summary, this study aimed to: (1) identify latent classes of cyberchondria among nursing interns using latent profile analysis; (2) examine the associations between work stress and membership in these latent classes; and (3) test the mediating effect of health anxiety in the relationship between work stress and latent classes of cyberchondria. The research hypotheses were as follows: (1) Cyberchondria among nursing interns is heterogeneous and can be classified into distinct latent classes; (2) Work stress has differential associations with membership across these latent classes; (3) Health anxiety mediates the relationship between work stress and latent classes of cyberchondria, and the effect of this mediation varies across classes.

## 2 Methods

### 2.1 Study setting and participants

A convenience sampling method was used to recruit nursing interns from 14 tertiary Grade-A hospitals in Hubei Province between September and November 2025 as study participants. Inclusion criteria were: (1) full-time nursing students currently undergoing clinical internship; (2) internship duration of 3–5 months (excluding pre-job training), with the internship still ongoing at the time of the survey; and (3) provision of written informed consent and voluntary participation in the study. Exclusion criteria were: (1) a diagnosis of a mental disorder—such as anxiety disorder, depressive disorder, or obsessive-compulsive disorder—within the past 6 months; (2) presence of a serious physical illness that impairs normal internship participation or information processing; and (3) interruption of the internship for 2 weeks or longer due to reasons including sick leave or suspension of studies during the internship period. This study was approved by the Ethics Committee of Hubei University of Chinese Medicine (approval number: 2025059) prior to data collection.

### 2.2 Sample size

Based on previous studies on sample size requirements for latent profile analysis (22, 23), at least 400 participants are needed to obtain a robust model fit. On this basis, to provide a quantitative reference for the required sample size, we used the formula for estimating the population mean in cross-sectional studies: $n={\left(\frac{\mu_{\alpha }\sigma }{\delta }\right)}^{2}$ (24), where the significance level α was set at 0.05, with μ_α_ = 1.96, the allowable margin of error δ was 0.06, and the standard deviation σ of the Chinese version of the Short Cyberchondria Scale—estimated from a pilot study (*n* = 50)—was 0.62. The calculated minimum required sample size was 410 participants. Accounting for a 20% anticipated rate of invalid questionnaires, the target sample size was increased to at least 492 participants. Ultimately, 538 participants were enrolled in this study.

### 2.3 Data collection instruments

#### 2.3.1 Demographic characteristics

Based on a literature review (25, 26) and discussions within the research team, a self-designed general information questionnaire was developed, covering age, gender, educational level, place of origin, internship duration, current internship department, health status, and the frequency and duration of recent online health information browsing.

#### 2.3.2 Chinese version of the short cyberchondria scale

This scale was developed by McElroy et al. (27) and translated into Chinese by Jin et al. (4). It comprises 12 items organized across four dimensions: excessive concern, negative interference, help-seeking behavior, and search anxiety. A 5-point Likert scale is used, with response options ranging from “strongly disagree” to “strongly agree”; higher scores indicate greater severity of cyberchondria. There are no reverse-scored items. In this study, the Cronbach's α coefficient for the scale was 0.938.

#### 2.3.3 Chinese version of the short health anxiety inventory

This scale was originally developed by Salkovskis et al. (11) in 2002, and was translated into Chinese and validated among Chinese university students by Zhang et al. (28). It consists of 18 items, each rated on a 4-point Likert scale ranging from 0 to 3. A total score of 15 or higher on the Short Health Anxiety Inventory (SHAI) indicates clinically significant health anxiety; higher scores reflect greater severity of health anxiety. In this study, the Cronbach's α coefficient for the scale was 0.949.

#### 2.3.4 Nursing internship stress scale

This scale was developed by Xiao et al. (29). It consists of 37 items across six dimensions: work nature, workload, internship preparation, interpersonal relationships, work support, and conflict between study and work. Each item is rated on a 4-point scale from 0 to 3, representing “no stress” to “severe stress”, with higher scores indicating greater perceived stress. Stress levels were classified into three categories based on the mean item score: low stress (mean <1.00), moderate stress (1.01–2.00), and high stress (2.01–3.00). In this study, the Cronbach's α coefficient for the scale was 0.968.

### 2.4 Data collection

Data were collected using the Wenjuanxing platform. The first page of the electronic questionnaire contained standardized instructions explaining the study's purpose, precautions, anonymous data processing, and the principle of full confidentiality. Nursing interns voluntarily completed the questionnaire after providing informed consent. To ensure data quality, all questions were set as mandatory. IP address tracking was applied to flag potential duplicate submissions for subsequent manual verification, rather than to automatically reject submissions at the front end; this design prevented the inappropriate exclusion of legitimate responses from participants sharing hospital public network environments (e.g., network address translation, NAT). The collected questionnaires were reviewed on a daily basis. Invalid responses were defined as those with eight or more consecutive identical answers, a completion time of less than 3 min, or confirmed duplicate submissions after multi-dimensional verification (demographic characteristics, response patterns, and item consistency), and these were excluded from the final analysis. A total of 559 questionnaires were initially collected. After quality screening, 21 invalid questionnaires were excluded, and 538 valid questionnaires were finally included in the analysis, corresponding to an effective response rate of 96.2%.

### 2.5 Data analysis

Descriptive statistics and Pearson correlation analysis were performed using SPSS version 27.0. Normally distributed continuous variables were expressed as mean and standard deviation, while non-normally distributed continuous variables were expressed as median and interquartile range [M (P25, P75)]. Categorical variables were expressed as frequencies and percentages (%). Differences in general characteristics across cyberchondria categories among nursing interns were examined using either the chi-square test or Fisher's exact test, as appropriate. Latent profile analysis was conducted using Mplus version 8.3. Model fit indices included the log-likelihood (LL), Akaike information criterion (AIC), Bayesian information criterion (BIC), adjusted BIC (aBIC), Lo-Mendell-Rubin adjusted likelihood ratio test (LMRT), Bootstrap likelihood ratio test (BLRT), as well as entropy. Among these indices, lower AIC, BIC, and aBIC values indicate better model fit. The LMRT and BLRT compare the fit of models with k versus k−1 latent classes; a *P*-value <0.05 indicates that the k-class model fits significantly better than the (k−1)-class model (30). An entropy value ≥ 0.8 suggests classification accuracy ≥ 90%. All mixture models were estimated with 200 random starts and 50 final-stage optimizations to avoid local maxima and ensure convergence to the global optimal solution. Sensitivity analyses were conducted using subscale-level indicators to verify the robustness of the latent profile structure. Three-step sensitivity analyses (R3STEP and BCH) were also performed in Mplus 8.3 to assess the impact of classification uncertainty on covariate and mediation effect estimates (31, 32). Structural equation modeling was performed using Amos version 31.0 to examine the mediating effect of health anxiety between work stress and latent classes of cyberchondria among nursing interns. The significance level was set at α = 0.05.

## 3 Results

### 3.1 Common method bias

Data in this study were collected through self-report measures—a method prone to common method bias, which may undermine the credibility of subsequent analytical results (33). Therefore, Harman's single-factor test was conducted to assess common method bias across all scale items. A total of 10 factors with eigenvalues greater than 1 were extracted, and the variance explained by the first factor was 33.36%, below the conventional 40% threshold. This suggests that common method bias does not pose a serious threat to the validity of the findings in this study.

### 3.2 Sample characteristics

Among the 538 nursing interns, ages ranged from 19 to 22 years. There were 68 males and 470 females. In terms of educational level, 322 were undergraduate students and 216 were junior college students. Regarding place of origin, 348 were from urban areas and 190 from rural areas. With regard to health status, 245 reported being healthy and 293 reported being in a sub-healthy state. Regarding the frequency of recent online health information browsing, 352 participants browsed such information 1–3 times per month, 156 browsed it 1–3 times per week, and 30 browsed it 1–3 times per day. With respect to the duration of each online health information session, 286 participants spent less than 10 min, 227 spent 10–30 min, and 25 spent more than 30 min. In terms of health anxiety, 366 participants (68.0%) met the cutoff for clinically significant health anxiety (SHAI total score ≥ 15).

### 3.3 Pearson's analysis of the correlation between cyberchondria, work stress, and health anxiety among nursing interns

Cyberchondria showed significant positive correlations with both health anxiety and work stress (all *P* < 0.05). Health anxiety also showed a significant positive correlation with work stress. Detailed results are presented in Table 1.

**Table 1:** Pearson's analysis of the correlation between cyberchondria, work stress, and health anxiety among nursing interns.

Variables	Mean	SD	Range	Cyberchondria	Health anxiety	Work stress
Cyberchondria	3.41	0.81	1.00–5.00	1		
Health anxiety	1.05	0.62	0.00–3.00	0.480^***^	1	
Work stress	1.30	0.56	0.00–3.00	0.287^***^	0.420^***^	1

^***^P < 0.001.

Values in the table are item mean scores. SD = standard deviation.

### 3.4 Latent profile analysis of cyberchondria among nursing interns

In this study, scores on the 12 items of the Chinese version of the Short Cyberchondria Scale were used as manifest variables to identify latent profile classes of cyberchondria among nursing interns. Starting with a one-class model (Model 1) as the baseline, models with increasing numbers of classes were estimated, resulting in a series of models ranging from one to five classes (see Table 2). Entropy values for all models exceeded 0.8, indicating adequate classification accuracy. As the number of classes increased, AIC, BIC, and aBIC values decreased gradually. The Lo-Mendell-Rubin adjusted likelihood ratio test (LMRT) for the five-class model was nonsignificant, suggesting that adding a fifth class did not significantly improve model fit beyond the four-class model. The average posterior probabilities (AvePP) for the four classes were 0.981, 0.964, 0.949, and 0.969, respectively, with corresponding odds of correct classification (OCC) all greater than 18, indicating high classification accuracy. The smallest class comprised 47 participants (8.7% of the total sample), satisfying the sample size requirement for stable latent profile estimation. Sensitivity analyses at the subscale level further verified the stability of the core hierarchical structure of cyberchondria profiles. Considering both statistical fit indices and substantive interpretability, the four-class model was selected as the optimal solution.

**Table 2:** Model fit indices for latent profiles of cyberchondria among nursing interns.

Model	LL	AIC	BIC	aBIC	Entropy	LMRT (*P*)	BLRT (*P*)	Class proportions (%)
1	−9,446.047	18,940.094	19,043.002	18,966.818				
2	−8,437.256	16,948.513	17,107.164	16,989.713	0.888	<0.001	<0.001	50.7%/49.3%
3	−7,773.353	15,646.707	15,861.100	15,702.383	0.938	<0.001	<0.001	9.0%/34.9%/56.1%
**4**	-**7,456.406**	**15,038.813**	**15,308.948**	**15,108.964**	**0.934**	**<0.001**	**<0.001**	**8.7%/44.2%/9.1%/38.0%**
5	−7,270.222	14,692.445	15,018.322	14,777.072	0.931	0.2585	<0.001	7.4%/22.3%/32.5%/29.1%/8.7%

Bold type indicates the optimal 4-class latent profile model selected for this study. LL, Log-likelihood; AIC, Akaike information criterion; BIC, Bayesian information criterion; aBIC, adjusted BIC; LMRT, Lo-Mendell-Rubin adjusted likelihood ratio test; BLRT, Bootstrap likelihood ratio test.

**Figure 1:**
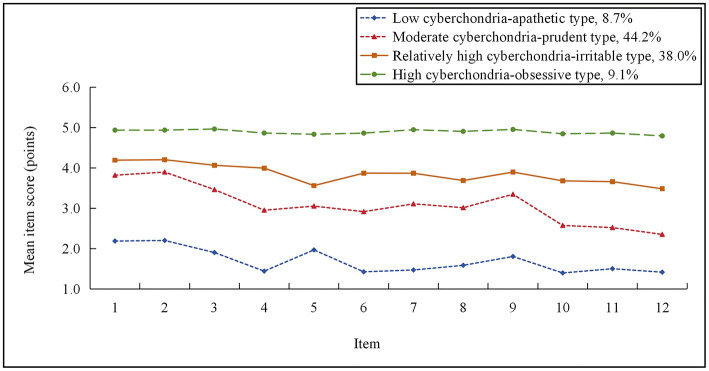
Estimated mean item scores for the four latent profiles of cyberchondria among nursing interns.

Based on Model 4, the mean scores of each item of the Cyberchondria Scale across the four latent classes are presented in Figure 1. The four classes were named based on the fluctuation patterns of the item mean scores, as well as the professional attributes and clinical internship characteristics of the nursing interns. Class 1 (C1) consisted of 47 participants (8.7%), with mean item scores below 2.0. This suggests that nursing interns in this class paid little attention to online health information, exhibited mild health-related concerns, and displayed apathetic cyberchondria-related behaviors. Consequently, this class was labeled “the low cyberchondria-apathetic” type. Class 2 (C2) consisted of 238 participants (44.2%), with mean item scores ranging from 2.0 to 3.5. This indicates that nursing interns in this class paid moderate attention to online health information, carefully evaluated health risks, and exhibited prudent cyberchondria-related behaviors. Therefore, this class was labeled “the moderate cyberchondria-prudent” type. Class 3 (C3) comprised 204 participants (38.0%), with mean item scores ranging from 3.5 to 4.0. This indicates that nursing interns in this class paid relatively close attention to online health information, were prone to emotional fluctuations in response to health-related content, and exhibited irritable cyberchondria-related behaviors. Therefore, this class was labeled “the relatively high cyberchondria-irritable” type. Class 4 (C4) comprised 49 participants (9.1%), with mean item scores exceeding 4.5. This indicates that nursing interns in this class paid excessive attention to online health information, repeatedly checked their health status, and exhibited obsessive cyberchondria-related behaviors. Therefore, this class was labeled the “high cyberchondria-obsessive” type.

### 3.5 Univariate analysis of different latent classes of cyberchondria among nursing interns

The results showed no statistically significant differences in latent cyberchondria classes among nursing interns across age groups, educational levels, places of origin, internship durations, or internship departments (all *P* > 0.05). Variables associated with statistically significant differences are presented in Table 3.

**Table 3:** Univariate analysis of different latent classes of cyberchondria among nursing interns.

Characteristics	Low cyberchondria–apathetic type N (%)	Moderate cyberchondria–prudent type N (%)	Relatively high cyberchondria–irritable type N (%)	High cyberchondria–obsessive type N (%)	Total N (%)	*χ^2^*
Gender						8.598^*^
Male	8 (11.8%)	24 (35.3%)	24 (35.3%)	12 (17.6%)	68 (12.6%)	
Female	39 (8.3%)	214 (45.5%)	180 (38.3%)	37 (7.9%)	470 (87.4%)	
Health status						7.904^*^
Healthy	30 (12.2%)	106 (43.3%)	85 (34.7%)	24 (9.8%)	245 (45.5%)	
Sub–healthy	17 (5.8%)	132 (45.1%)	119 (40.6%)	25 (8.5%)	293 (54.5%)	
Frequency of recent online health information browsing						16.813^**^
1–3 times per month	38 (10.8%)	161 (45.7%)	119 (33.8%)	34 (9.7%)	352 (65.4%)	
1–3 times per week	8 (5.1%)	65 (41.7%)	74 (47.4%)	9 (5.8%)	156 (29.0%)	
1–3 times per day	1 (3.3%)	12 (40.0%)	11 (36.7%)	6 (20.0%)	30 (5.6%)	
Duration of each online health information session						16.677^**^
<10 min	33 (11.5%)	128 (44.8%)	98 (34.3%)	27 (9.4%)	286 (53.2%)	
10–30 min	10 (4.4%)	102 (44.9%)	98 (43.2%)	17 (7.5%)	227 (42.2%)	
>30 min	4 (16.0%)	8 (32.0%)	8 (32.0%)	5 (20.0%)	25 (4.6%)	

^*^P < 0.05; ^**^P < 0.01.

### 3.6 The mediating role of health anxiety between work stress and latent profiles of cyberchondria among nursing interns

**Figure 2:**
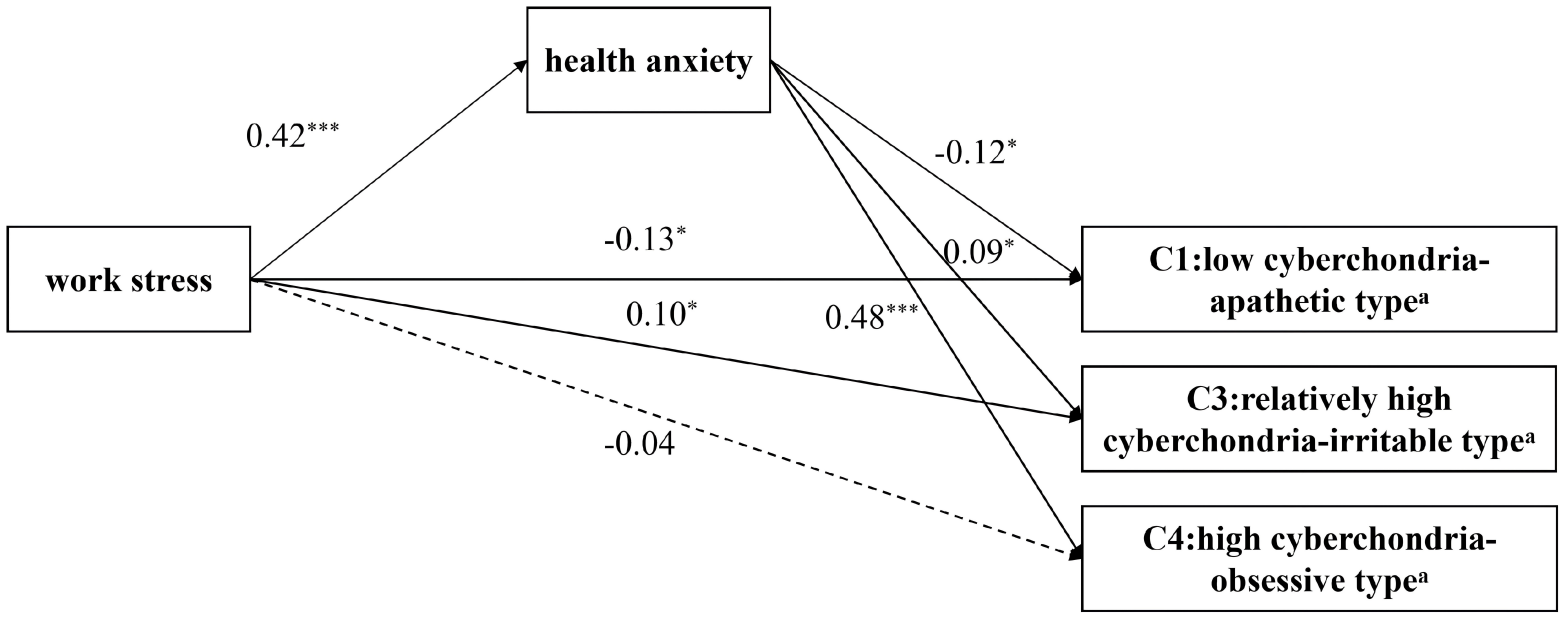
Mediating path diagram of health anxiety in the relationship between work stress and cyberchondria. “a” denotes the moderate cyberchondria-prudent type as the reference group. *P < 0.05, ***P < 0.001. All latent classes of cyberchondria among nursing interns were dummy-coded.

After controlling for gender, health status, frequency of recent online health information browsing, and duration of each online health information session, we constructed a structural equation model with work stress score as the independent variable, health anxiety score as the mediator, and the three latent classes of cyberchondria (using the moderate cyberchondria-prudent type as the reference group) as the dependent variable to test the mediating effect of health anxiety. The model's path estimates are shown in Figure 2. Work stress score was positively associated with health anxiety score (β = 0.42, *P* < 0.001). Health anxiety score was negatively associated with the low cyberchondria-apathetic class (β = −0.12, *P* = 0.023) and positively associated with both the relatively high cyberchondria–irritable class (β = 0.09, *P* = 0.027) and the high cyberchondria-obsessive class (β = 0.48, *P* < 0.001). Work stress score was negatively associated with the low cyberchondria-apathetic class (β = −0.13, *P* = 0.019), positively associated with the relatively high cyberchondria–irritable class (β = 0.10, *P* = 0.037), and showed no statistically significant association with the high cyberchondria–obsessive class (β = −0.04, *P* = 0.382).

Bootstrap tests revealed that health anxiety exhibited significant indirect associational pathways between work stress and three latent classes of cyberchondria: the low cyberchondria-apathetic type (standardized effect = −0.026, *P* < 0.05), the relatively high cyberchondria-irritable type (standardized effect = 0.034, *P* < 0.05), and the high cyberchondria-obsessive type (standardized effect = 0.105, *P* < 0.001) (see Table 4). Among these, health anxiety fully mediated the association between work stress and the high cyberchondria-obsessive type, whereas it partially mediated the associations with the other two types. Moreover, the standardized indirect effect of health anxiety in the relationship between work stress and the high cyberchondria-obsessive type was significantly larger than those for the other two types, indicating a differentiated mediating role of health anxiety in how work stress influences distinct latent profiles of cyberchondria.

**Table 4:** Mediation analysis of health anxiety between work stress and latent classes of cyberchondria among nursing interns.

Class	Path	β	95%CI	
Low cyberchondria–apathetic type	Work stress → Low cyberchondria–apathetic type	−0.128	−0.226	−0.024
	Work stress → Health anxiety → Low cyberchondria-apathetic type	−0.026	−0.049	−0.005
	Total effect	−0.091	−0.143	−0.040
Relatively high cyberchondria-irritable type	Work stress → Relatively high cyberchondria-irritable type	0.104	0.008	0.200
	Work stress → Health anxiety → Relatively high cyberchondria-irritable type	0.034	0.004	0.071
	Total effect	0.125	0.046	0.198
High cyberchondria-obsessive type	Work stress → High cyberchondria-obsessive type	−0.043	−0.130	0.052
	Work stress → Health anxiety → High cyberchondria-obsessive type	0.105	0.074	0.143
	Total effect	0.082	0.032	0.139

The moderate cyberchondria-prudent type served as the reference group.

## 4 Discussion

### 4.1 Nursing interns exhibit significant group heterogeneity in cyberchondria

The results of this study showed that the mean item score of cyberchondria among nursing interns was 3.41 (SD = 0.81), which was higher than that of the general university student population (4, 28). This difference may be attributed to the unique stressors faced by nursing interns, such as prolonged exposure to clinical healthcare settings and direct confrontation with occupational health risks. Consistent with findings from pre-clinical undergraduate nursing student cohorts (18), the overall cyberchondria level in this intern sample was slightly elevated, which likely reflects the effect of increased clinical exposure: unlike pre-clinical students with limited direct patient contact, interns are routinely exposed to patient illness narratives, occupational biohazard risks and high-pressure shift environments, which may heighten baseline health concern and increase vulnerability to cyberchondria. The four-profile structure identified here is broadly congruent with the three-tier classification reported in non-intern nursing students, while the additional distinction between the moderate prudent subtype and relatively high irritable subtype may reflect the unique stress profile of trainees navigating direct clinical practice. Based on latent profile analysis, four latent classes of cyberchondria were identified among nursing interns: the low cyberchondria–apathetic class, the moderate cyberchondria-prudent class, the relatively high cyberchondria-irritable class, and the high cyberchondria-obsessive class—indicating significant heterogeneity in cyberchondria among nursing interns. Specifically, the moderate cyberchondria-prudent type accounted for the highest proportion (44.2%). This finding is closely related to the professional background of nursing interns, who have systematically acquired medical knowledge and developed preliminary skills in symptom differentiation. As a result, they neither ignore health information nor uncritically accept extreme online health descriptions. The relatively high cyberchondria–irritable class ranked second (38.0%). Nursing interns in this class were prone to emotional fluctuations triggered by online health information. This characteristic may be linked to their exposure to clinical stress: when rotating through high-pressure departments—such as emergency departments or intensive care units—they frequently encounter critically ill patients and invasive procedures, face potential occupational exposure risks, and experience self-blame following procedural errors (34). These factors may continuously amplify concerns about their own health and reinforce health-related worries. The high cyberchondria-obsessive class accounted for a relatively low proportion (9.1%). Nursing interns in this class exhibited excessive attention to online health information and were at elevated risk for cyberchondria. This class may be associated with a higher baseline level of health anxiety. In the clinical context, some nursing interns in this group may have experienced negative health-related events during their internship, which could contribute to the generalization of health concerns and reinforce a cycle of repeated online health searching and increased anxiety (12). The low cyberchondria-apathetic class accounted for the smallest proportion (8.7%). Nursing interns in this cohort exhibited mild cyberchondria. This may be attributable to their strong professional confidence and heightened health awareness, which support rational differentiation between normal physical discomfort and disease-related symptoms—reducing reliance on online information for health status confirmation. Notably, the 87.4% female proportion in this sample closely mirrors the actual gender structure of China's nursing workforce, where long-standing cultural gender norms frame caregiving work as congruent with traditionally feminine traits of meticulousness and empathy (35). In line with latent profile evidence from Chinese undergraduate nursing student samples, gender shows a significant association with cyberchondria profile membership (18). Female trainees are more likely to be classified into higher-symptom cyberchondria profiles, while male trainees are overrepresented in the low-symptom group. One possible explanation is that women in frontline clinical settings take on a larger share of emotional care labor, which may increase sensitivity to health-related threats. A second related factor is gendered socialization norms, which tend to encourage closer attention to bodily symptoms and health information among women. Research indicates that providing targeted psychological support alongside health information literacy education is an effective strategy for reducing cyberchondria risk (36, 37). Therefore, nursing educators and clinical administrators should implement stratified interventions, including facilitating the development of adaptive emotion regulation mechanisms among nursing interns through systematic stress management training and instruction in evidence-based stress reduction techniques, thereby indirectly mitigating internet-related health anxiety; and enhancing critical appraisal skills related to health information, including guidance on identifying misinformation and exaggerated claims, avoiding unnecessary health anxiety triggered by unreliable content, and reinforcing the capacity to evaluate health information critically.

### 4.2 Associations between work stress and membership in different cyberchondria classes exhibits directional differences

This study found that the associations between work stress and membership in different cyberchondria classes among nursing interns varied directionally, indicating that the influence of work stress on cyberchondria is not a single linear effect. Specifically: (1) Work stress was significantly and positively associated with membership in the relatively high cyberchondria-irritable class, suggesting that work stress is an important risk factor for this class. The work stressors encountered by nursing interns during clinical practice—such as heavy workloads, operational risks, and occupational exposure—continuously heighten their concerns about personal health, thereby increasing their susceptibility to emotional fluctuations triggered by online health information (20). This finding further refines the conceptualization of the relationship between work stress and psychological sensitization among nursing interns. (2) Work stress was significantly and negatively associated with membership in the low cyberchondria-apathetic class, indicating that higher work stress among nursing interns was associated with a lower probability of classification into this class. A possible explanation is that stress activates health-related concerns, prompting nursing interns in this class to shift from neglecting health information to actively monitoring their own health status. This shift represents a typical stress-triggered self-protective behavior (38, 39). (3) The direct association between work stress and the high cyberchondria-obsessive class was not statistically significant, suggesting that membership in this latent class arises from multiple interacting factors, rather than being directly triggered by work stress. Further investigation into the mediating roles of psychological variables—such as health anxiety—is warranted (13).

### 4.3 Health anxiety plays a differentiated mediating role between work stress and latent profiles of cyberchondria

Previous research has shown that high levels of health anxiety drive individuals to search for health information online more frequently, which is in turn associated with elevated anxiety and worry (12, 40). This anxiety not only directly prompts online health information-seeking behavior among nursing interns but also reinforces cyberchondria through a vicious cycle of “searching-anxiety exacerbation” (10). Health anxiety plays a differential mediating role between work stress and distinct cyberchondria profiles among nursing interns, reflecting population heterogeneity. (1) In terms of mediation type, health anxiety played a full mediating role in the high cyberchondria-obsessive type, whereas it played a partial mediating role in the low cyberchondria-apathetic and relatively high cyberchondria-irritable types. In this study, the obsessive type emerged as work stress continuously heightened health anxiety, prompting nursing interns to repeatedly search for health-related information in an attempt to alleviate that anxiety—ultimately resulting in obsessive behavior. In contrast, the apathetic and irritable types arose from the combined effects of work stress (direct effect) and health anxiety (mediating effect). (2) In terms of the direction and magnitude of the mediating effects, health anxiety exerted a negative mediating effect on the low cyberchondria-apathetic type, a positive mediating effect on the relatively high cyberchondria-irritable type, and a positive mediating effect on the high cyberchondria-obsessive type. Moreover, the magnitude of the mediating effect was significantly greater for the obsessive type than for the other two types—a difference that may be attributable to baseline differences in health anxiety levels across nursing intern types. Specifically, the apathetic type exhibited a relatively low baseline level of health anxiety; consequently, work stress induced only a modest increase in health anxiety among these individuals, who also demonstrated greater confidence in their own health status. As a result, they displayed an “apathetic” orientation toward health-related information and showed little reliance on online sources to verify potential health risks. In contrast, the irritable and obsessive cyberchondria subtypes exhibited relatively high baseline levels of health anxiety and were more sensitive to perceived work stress. The increase in health anxiety triggered by work stress amplified their cyberchondria behaviors. Moreover, the obsessive type showed more pronounced generalization of health anxiety, resulting in a stronger mediating effect. Furthermore, the professional characteristics of nursing interns provide a population-level basis for this differential mediation: their foundational medical knowledge is insufficient to support accurate differential diagnosis of diseases; consequently, driven by health anxiety, they tend to overinterpret online health information—a tendency that further reinforces their cyberchondria behaviors. This also explains why health anxiety plays a significant mediating role between work stress and cyberchondria (41).

### 4.4 Limitations

This study has several limitations. First, the cross-sectional design can only reveal associations among variables and cannot establish causal relationships. Second, the sample was limited to nursing interns from 14 tertiary Grade-A hospitals in Hubei Province, which may restrict the generalizability of the findings due to the narrow geographical scope. Third, while the anonymous survey design precluded direct calculation of hospital-level intraclass correlation coefficients (ICCs), we conducted department-level sensitivity analyses as a conservative proxy. The estimated department-level ICCs were negligible, and results remained virtually unchanged under cluster-robust adjustment. Fourth, this study did not include measures of eHealth literacy, digital health competency, or general anxiety and depression. Existing evidence has confirmed that these factors are closely associated with the formation and development of cyberchondria (42). The omission means that we cannot fully disentangle the specific effect of health anxiety from the influence of general negative affect, nor can we further identify the underlying drivers of different latent profiles, which to some extent restricts the depth of causal inference and subtype interpretation. Fifth, the proportion of female nursing interns in the sample was as high as 87.4%, indicating a marked gender imbalance; this may hinder the characterization of cyberchondria among male nursing interns and thus reduce the representativeness of the results for the broader population of nursing interns. Finally, all data were collected via self-report measures, which may introduce social desirability bias and response style artifacts. Although distributional checks confirmed no significant ceiling effects and Harman's single-factor test indicated no severe common method bias, potential response biases cannot be fully eliminated, which may compromise data objectivity to some extent.

## 5 Conclusion

This study identified four latent classes of cyberchondria among nursing interns using latent profile analysis, revealing substantial heterogeneity within this population. Work stress showed directional differences in its associations with membership across these cyberchondria classes, and health anxiety played a differential mediating role in these associations. Therefore, nursing educators and clinical administrators should acknowledge the heterogeneity of cyberchondria in nursing interns and implement stratified intervention strategies—targeting both work stress reduction and tailored health anxiety management—to effectively mitigate cyberchondria. Future multicenter, large-sample longitudinal studies with more gender-balanced participant compositions are warranted to further elucidate the dynamic causal pathways linking work stress, health anxiety, and cyberchondria, and to verify potential gender differences in cyberchondria profiles among nursing interns. In particular, incorporating variables such as eHealth literacy and general emotional distress will help clarify the differentiated driving mechanisms of different cyberchondria subtypes and verify the stability of the mediating pathway under more rigorous confounding control, so as to provide more robust evidence for the development of precise stratified intervention protocols.

## Data Availability

The original contributions presented in the study are included in the article/supplementary material, further inquiries can be directed to the corresponding author.
